# Socio-Cultural Beliefs, Values and Traditions Regarding Women’s Preferred Mode of Birth in the North of Iran

**Published:** 2015-07

**Authors:** Robab Latifnejad Roudsari, Maryam Zakerihamidi, Effat Merghati Khoei

**Affiliations:** 1 Evidence-Based Care Research Centre, Department of Midwifery, Mashhad University of Medical Sciences, Mashhad, Iran;; 2 Department of Midwifery, School of Medicine, Tonekabon Branch, Islamic Azad University, Tonekabon, Iran;; 3 Iranian National Center for Addiction Studies (INCAS), Institution of Risk Behavior Reduction, Tehran University of Medical Sciences, Tehran, Iran

**Keywords:** Birth, Cultural beliefs, Iran, Qualitative study, Women

## Abstract

**Background:**

Pregnant women rely heavily on informal information while making a decision about the mode of delivery they would rather have, either as normal vaginal delivery (NVD) or cesarean section (CS). Through recognition of social attitudes towards different modes of delivery, societies can be directed towards a positive understanding of vaginal delivery, which can ultimately lead to maternal health promotion. Thus, this study aimed to explore the common beliefs, values and traditions surrounding women’s preferred mode of birth in the North of Iran.

**Methods:**

Using a focused ethnographic approach, twelve pregnant women, 10 women with previous experience of childbirth, seven midwives, seven obstetricians, and nine non-pregnant women were included in this study through a purposeful sampling in health clinics of Tonekabon in the North of Iran. Semi-structured interviews and participant observations were used for data collection. Study rigor was confirmed through prolonged engagement, member check, expert debriefing, and thick description of the data. Data were analysed using Braun & Clarke thematic analysis (2006) and MAXqda software.

**Results:**

Through analysis, three major themes and 10 subthemes emerged.  They included: 1) sociocultural childbirth beliefs with five subthemes: a) CS as protector of genital tract integrity, b) blind imitation in choosing mode of birth, c) NVD as a low cost type of delivery,  d) CS as a prestigious mode of birth and, e) NVD as a symbol of woman’s power and ability; 2) traditional health beliefs with two subthemes: a) NVD as a guarantee for woman’s health, b) traditional childbirth facilitators; 3) religious beliefs and values with three subthemes: a) NVD as a symbol of God’s power, b) call for help from the Mighty God, and c) NVD as a sacred phenomenon.

**Conclusion:**

The results of this study indicated that cultural beliefs, values and traditions can significantly affect individuals’ attitudes towards modes of delivery, their definitions of different modes, and the decisions they make in this regard. In order to develop a positive cultural and religious attitude towards vaginal delivery, women’s awareness has to be raised through various ways and the existing misconceptions need to be corrected.

## Introduction

Pregnant women are normally involved in a decision-making process concerning the mode of delivery, and many factors affect their decisions.^1^ These processes are influenced by a person’s environment, values, personality, knowledge, and insight, which influence each other interactively.^2^

CS rate varies in different parts of the world and is rapidly rising in many countries.^3^ In U.S.A., C-section accounts for one in every 10 childbirths; in  2002, C-section rate increased to 26.1% of all deliveries.^4^ In Iran, according to official statistics, the incidence of C-section is on average three times higher than the global rate. In fact, the rate of C-section was 36% in 2002^5^ and 42.3% in 2006.^6^

Social science research indicates that one of the main sources of information for selecting the mode of delivery is the account of experiences mothers hear from other women in social gatherings and gabfests.^7^ These “birth stories” are mostly concerned with unpleasant aspects of childbirth, such as physical pain, psychological pressure at the time of delivery, inappropriate midwifery interventions, and emergencies. These negative aspects are also emphasized by social media. Considering the negative effects of these outlooks on women, some opt for C-section only because it is a depiction of high social class or a “trend” in the society.^8^

In general, apart from personal and medical factors, a number of social and cultural issues are also involved in a pregnant woman’s tendency towards a certain mode of delivery. Today, C-section in most countries seems to be the first option for pregnant women.^9^^,^^10^

There are many questions regarding the effects of personal, religious, and traditional health beliefs on one’s preference for a mode of delivery. These questions need to be addressed in the cultural context of Iran. Most women in the city of Tonekabon consider vaginal delivery as a painful and exhausting experience, followed by very few complications. On the other hand, C–section is considered a painless and simple procedure, which is usually preferred due to fear of labor pain during vaginal delivery. Few qualitative studies have been conducted in Iran to explore the conditions which influence women’s cultural beliefs towards mode of birth. Latifnejad Roudsari et al. (2014), in a focused ethnographic study, found that cultural norms and values and social network are the factors encouraging the choice of cesarean delivery.^11^ Also, Zakerihamidi et al. (2014), in a qualitative study, reported that considering childbirth as something sacred based on the participant’s religious beliefs affected the participants’ decision to choose vaginal delivery.^12^ They reported in a similar study (Zakerihamidi et al., 2015) that women perceived NVD as a facilitator of women’s physical and mental health promotion, and saw cesarean section as a painless mode of delivery.^13^

There is little knowledge of women’s beliefs about different modes of delivery, and no extensive studies have been conducted on this subject as a cultural phenomenon in Iran. Studies of the effects of cultural issues on women’s decisions about delivery mode can contribute to the formulation of policies to confront problems associated with C-section. As a result, this study, by applying an ethnographic approach, was conducted to explore the cultural beliefs, values and traditions of women towards modes of delivery in Tonekabon, in the North of Iran. 

## Materials and Methods

Since the current study aimed to explore cultural beliefs about modes of delivery, it was necessary to study social interactions, behavioral patterns, beliefs, and attitudes within groups of people. A focused or micro-ethnographic approach seemed appropriate for this purpose, as it was suitable for knowing cultural beliefs and customs relevant to the mode of delivery. A Focused ethnographic approach could provide an in-depth understanding of beliefs and traditions about different modes of delivery,^14^ and was deemed to be suitable for the current study. Focused ethnography is a qualitative method for studying a special culture or phenomenon by field work and record of cultural beliefs and behaviors. Ethnographers describe cultural members, phenomena and problems using culture as a lens. Focused ethnography emphasizes the importance of studying human behaviors in a culture to understand the cultural rules, norms and common practices.^8^ In this method, the main focus is on the dominant culture as well as the subcultures in a specific cultural field^15^ with a small sample.^16^ The results of such approach could be applied to adopt culture-oriented strategies. This research approach could also provide sufficient data in relation to the beliefs, values and cultural traditions about modes of delivery in order to help health professionals develop culture-based health promotion programs.^17^

Twelve pregnant women and 10 women with previous experience of delivery (postpartum women) participated in this study; moreover, seven midwives, seven obstetricians, and nine non-pregnant women were also interviewed at healthcare centers, hospitals, and gynecology clinics of Tonekabon, Iran, from January 2013 to September 2014.

Pregnant women in their third trimester who were referred to healthcare centers or clinics for prenatal care, as well as postpartum women who were hospitalized or referred for postpartum care to gynecology clinics or healthcare centers of Tonekabon (during 42-60 day after delivery) were included in the study. Additionally, in order to collect some complementary data, several midwives and obstetricians were interviewed.

Using the purposive sampling method and maximum variation strategy, the participants were selected from people who could provide accurate and rich data about the topic of the study. The sampling was done in various groups of pregnant women and women with previous experience of natural or cesarean delivery and non-pregnant women of different ages, education, jobs, gravidity and parity status. Also, midwives and gynecologists were selected from those who were experts in the related field of study or had at least five years of educational, research, and treatment experiences.

The researchers used the main methods of data collection in focused ethnography, such as observation, interview and documents, in a continuous process and returned to the field of study to conduct more interviews and observation.^18^ After assuring the participants about the confidentiality of the interviews, the researchers carried out 45 observations and semi-structured interviews in a private and relaxing environment in the healthcare centers, hospitals, and gynecology clinics of Tonekabon.

In general, the purpose of observation was the evaluation of people’s behaviors; this evaluation contributed to the description of the events in a setting.^19^ Observations were made as “observer as participant”, which is the selected method of observation in focused ethnography, and were recorded as field notes. In the present study, the researchers were resident in the health centers, hospitals, and delivery wards as observers and as staff midwives to provide care to pregnant women and those who underwent CS and or NVD. This means that the participants in the study received the researchers’ care during or after pregnancy and the researchers, at the same time, observed how they made decisions about mode of delivery.^20^ In this study three forms of descriptive, focused, and selective observations were used. During these observations, nine components of culture were taken into account, which were as follows: people, actions, tools, events, functions, time, objectives, physical characteristics of the location, and individual’s feelings.^21^ The researchers gathered some field notes during observation which could provide new insights for researchers in the field under study.^22^ The researchers used their field notes to document their own observation and to record the nonverbal behaviors of the participants and their relationship with those who provided health care services. In fact, in these notes there were issues that the researchers heard, observed, as well as thought about and experienced during and after observation. The researchers tried to provide a thick description of the events, observed people, all interactions with the participants as well as their own personal emotions and analytical thoughts in the field notes to maintain a reflexive stance.

An interview guide was used to do semi-structured interviews. The questions were related to the following subjects: 1) participants’ personal beliefs about childbirth, 2) how events, people, and actions influenced their beliefs about the mode of delivery and 3) barriers affecting their beliefs. In qualitative studies, repetition of previous data as well as not emerging new or relevant issues, is indicative of the adequacy of sampling. After interviewing 45 participants, data saturation was achieved.

In this study, data collection and data analysis were carried out simultaneously. 

Braun & Clarke thematic analysis (2006)^23^ and MAXqda software were used for the analysis of the data. The thematic analysis provided a flexible way to analyze the data. In general, the methods of qualitative analysis are divided into two groups. The first group originates from particular ontological and epistemological stances, whereas the second group is independent of special theoretical perspectives and could be applied in a broad range of qualitative methodologies. The thematic analysis belongs to the latter group of analytical methods, and can be applied as a flexible analytical tool for analysis of thick and sophisticated data.^23^ So, it has been used in several ethnographic studies.^24-26^

The Thematic analysis which was used in this study included six steps:^23^ step 1: familiarity with data, step 2: generating primary codes, step 3: searching for themes, step 4: reviewing the themes, step 5: defining and naming the themes, and step 6: preparing a report.

To analyze data, every interview was first read through word by word and codes were derived from the participants’ statements; the codes were written in the margin of the text. In the current study, 1340 initial codes were extracted, which were merged to 420 codes after similarities and overlaps were evaluated and identified. Finally, 20 sub-sub themes grouped into 10 sub-themes and three major themes.

The following measures were taken for the reliability of the data: long-term engagement with the participants during data collection, constant observations, using different methods for data collection, using exact descriptions, having the extracted codes and themes confirmed by an expert (who was experienced in qualitative research and was not involved in the study), member check, searching for contrary evidence and providing reflexive journal.

The current study was confirmed by the ethics committee of Mashhad University of Medical Sciences. Before conducting the study, written consent was obtained from all the participants. The participants voluntarily took part in the study, and were allowed to withdraw from the study at any point. 

## Results

The average age of the participants was 25.19±4.68 and most of them were housewives (80%). 

Through analysis, three major themes and 10 subthemes emerged. They included: 1) sociocultural childbirth beliefs with five subthemes: a) CS as protector of genital tract integrity, b) blind imitation in choosing mode of birth, c) NVD as a low cost type of delivery, d) CS as prestigious mode of birth and, e) NVD as a symbol of a woman’s power and ability; 2) traditional health beliefs with two subthemes: a) NVD as a guarantee for woman’s health, and b) traditional childbirth facilitators; and 3) religious beliefs and values with three subthemes: a) NVD as a symbol of God’s power, b) call for help from the Mighty God and, c) NVD as a sacred phenomenon (Table 1).

**Table 1:** Themes and sub-themes as emerged from the data

**Themes **	**Sub-themes**
Socio-cultural beliefs about childbirth	CS as protector of genital tract integrity
	Blind imitation in choosing mode of birth
	NVD as a low-cost type of delivery
	CS as prestigious mode of birth
	NVD as a symbol of woman’s power and ability
Traditional health beliefs	NVD as a guarantee for woman’s health
	Traditional childbirth facilitators
Religious beliefs and values	NVD as a symbol of God’s power
	Call for help from the Mighty God
	NVD as a sacred phenomenon

Through analysis of interviews and observations, three major themes emerged from the obtained data including “sociocultural beliefs about childbirth”, “traditional health beliefs”, and “religious beliefs and values”, which are discussed below.

*1- Socio-Cultural Beliefs about Childbirth*

*a- CS as Protector of Genital Tract Integrity*

The Participants believed that the main benefit of CS, in comparison with vaginal delivery, is to preserve the beauty of genitalia and its performance and also to preserve the strength of sexual pleasure:

“I knew that I won’t experience pain when I go for cesarean and it guarantees the health of my baby and most importantly I felt that with cesarean I would have no problem in my sexual intercourse so I chose the cesarean.” (woman who had had CS, 32 years old, bachelor’s degree, in favor of C-section). 

They believed that the main advantage of cesarean delivery is protection of genital system integrity and, as a consequence, lack of perineal relaxation caused by vaginal delivery, which affects sexual pleasure. Hence, mothers avoided this mode of delivery. As one participant pointed out:

“My husband tells me that if I have a natural birth, we will have sexual problems in future.” (Pregnant woman, 27 years old, associate’s degree, first pregnancy)

*b- Blind Imitation in Choosing Mode of Birth*

“Blind imitation” was another important factor that was referred to in the participants’ statements. This can be summarized in a statement by one of the participants:

"When I want to decide on the mode of delivery, C-section seems a better option because others have chosen it too. I am not any less than any of them.” (Pregnant woman, 24 years old, diploma, first pregnancy)

As an interview indicated, one of the obstetricians regarded “blind imitation” as the main reason for the prevalence of C-section and mentioned that pregnant women even imitate others in their choice of the type of anesthesia during C-section.

Pregnant women use gynecologists as role models and choose C- section. One of the participants, regarding her view in relation to the superiority of cesarean delivery, believed that as gynecologists prefer cesarean section so it seems that it is the best method:

“I know and heard that none of the physicians use NVD delivery, so cesarean is good.” (Pregnant woman, 20 years old, first pregnancy, diploma)

*c- NVD as a Low-Cost Type of Delivery*

Since C-section is more expensive than vaginal delivery, people believe that it is exclusive to the upper class and consider it “fancy”; in fact, they think it is much more reliable and of higher quality:

“If you spend money, doctors take care of you much better. So, I want to have c-section because it is more expensive.” (Pregnant woman, 20years old, graduate student, first pregnancy) 

One pregnant woman says that her only reason to select NVD was economic problems and not being able to pay the hidden and obvious expenses of C-section: 

“At first I decided to have C- section, but now we are in debt due to buying a new house and can’t afford cesarean expenses so I have to select vaginal delivery”. (Pregnant woman, 25 years old, first pregnancy, Bachelor’s degree)

*d- CS as a Prestigious Mode of Birth*

The prestige associated with C-section has become a common cultural belief and a social norm and plays a significant role in pregnant women’s decision-making process. C-section is known as the choice of wealthy people and a prestigious mode of delivery. It is in fact considered as a priority for some pregnant women. As one of the interviewees pointed out:

“I want to have c-section because it shows my high social class.” (Pregnant woman, 22 years old, diploma, second pregnancy)

Since cesarean delivery is expensive and is the first choice of the rich, some of the participants consider it as a cause to feel proud of oneself: 

“My colleagues boast about their money and say they had had C- section, so I want it too.” (Pregnant woman, 25 years old, diploma, in favor of C-section)

*e- NVD as a Symbol of a Woman’s Power and Ability*

Some pregnant women believed that enduring labor pain represents a woman’s power and ability, and strengthens maternal feelings and the bond between mother and child. On the other hand, C-section was considered as a kind of failure to undergo vaginal delivery. They believed that a woman who chooses vaginal delivery is a strong person and can overcome the pain of vaginal delivery.One participant said:

“In my opinion, natural birth shows that the mother has the strength to tolerate such pain and give birth to a lovely creature. But C-section means the mother is unable to tolerate pain.” (Pregnant woman, 32years old, bachelor’s degree, second pregnancy)

*2- Traditional Health Beliefs*

*a - NVD as a Guarantee for a Mother’s Health*

Many pregnant women believed that safety of mother and fetus is guaranteed during natural delivery; therefore, they accepted this mode of delivery despite the excruciating pain. Some pregnant women stated that through vaginal delivery, the toxins are eliminated from the body and the body regains its health. In this regard, one of the pregnant women said:

"Vaginal delivery is difficult, but all the toxins get out of your body, which is a good thing.”(Pregnant woman, 28 years old, diploma, first pregnancy)

Similarly, some of the interviewed midwives confirmed that natural birth maintains maternal health, detoxifies the body, and strengthens the body’s defense mechanism. On the other hand, blood clots at the incision site can cause infection, pain, or disease for the mother. As one midwife remarked:

“During natural birth, the whole body is detoxified and all blood clots are removed from the body.” (A local midwife, 55 years old, BS, 20 years of work experience)

*b- Traditional Childbirth Facilitators*

Some pregnant women mentioned the use of cyclamen (a plant which grows in Mecca, Saudi Arabia) for an easy natural birth. One of the participants had even brought it with her to the hospital. She said that in the past, people used to put cyclamen in water until it blossomed. When the flower blossomed completely, natural birth was performed. She ascribed cyclamen to Mary and considered it as the agent for fetal development in her womb.

“I put an un-blossomed cyclamen in water; it starts to grow gradually. When it blossoms fully, the baby is delivered.” (Pregnant woman, 22 years old, diploma, before undergoing natural delivery)

Based on the observations of and interviews with the participants, some of the pregnant women who had decided to have vaginal delivery had prepared to cope with labor pain. In the different groups, the participants mentioned different foods, including saffron, borago officinalis, milk, date, sugarplum, honey, traditional foods, and castor oil for soothing labor pain. Also, some activities, including daily bathing, physical activity, hiking, carrying heavy objects before labor, and pain management at home prior to delivery, were introduced as facilitators for reducing the pain and shortening its period. In this regard, an interviewee remarked:

“If I eat saffron or drink some brewed borage with sugarplum or something like that, I can have a faster and easier delivery, since these foods are refreshing.” (28 years old, high-school diploma, first pregnancy)

*3. Religious Beliefs and Values*

*a -NVD as a Symbol of God’s Power*

Some of the interviewed pregnant women, postpartum women, and obstetricians believed that natural birth is a natural phenomenon, a symbol of God’s power, a divine gift which is not endowed to everyone, and a means of finding God. Conversely, C-section was considered an intervention in a natural process. As one of the participants said:

"Vaginal delivery is birth of a baby in the way that God intended it.”(A 34-year-old participant with previous experience of vaginal delivery, MSc student, second pregnancy)

Some gynecologists described vaginal delivery as a depiction of God’s power and glory. In this regard, one of the gynecologists stated:

“When I see a baby being born through the birth canal, the world is reborn for me. But I don’t have the same feeling during a cesarean delivery; I mean the baby comes much faster. Natural birth inspires one of the most beautiful feelings I’ve ever had. I always pray to God when I see the birth of a baby.” (Gynecologist, 48 years old, work experience: 18 years)

*b- Call for Help from the Mighty God*

The participants (pregnant and postpartum women) also suggested some strategies to cope with delivery pain. These recommendations were important in increasing pain tolerance and boosting psychological and spiritual strength during delivery; praying, praising God, promising offerings to God, and recourse to ‘Ahlulbayt’ were a few of them. Some of the pregnant women and midwives believed in tying written prayers like ‘Ayatolkorsi’ around the foot of an expecting mother before natural birth. This, they presumed, is a call for help from the Mighty God, which facilitates and thus advances the delivery. As one of the participants said:

“I write something and tie it around my foot. I believe it relieves my pain. It’s Ayatolkorsi.” (A 29-year-old woman, BS, second pregnancy)

One of the midwives believed that recourse to God is important for mothers since it helps them build up a positive attitude and feel supported. She stated that delivery progress is much easier among religious and traditional women compared to others. As one of the midwives said:

“Those who go for conventional and traditional outlets give birth much more easily.” (A 48-year-old midwife, BS, work experience: 25 years)

*c- NVD as a Sacred Phenomenon*

As the analysis of the interviews demonstrated, some of the pregnant women believed that natural birth is a holy phenomenon since one can praise God and get closer to Him. They believed that if the expecting mother died while giving birth, she could reach the sublime degree of martyrdom. The interviewees believed that tolerating labor pain results in the forgiveness of their sins:

“Natural birth is a way of approaching God. God forgives your sins when you experience such pain. If your fate is to die during labor, you will be a martyr” (A 27-year-old pregnant woman, diploma, previous experience of natural birth).

During the observation and interviews, most of the participants in different groups had special beliefs about normal delivery and believed that when women endured hard labor pain they would be free from sin, therefore their prayer will be accepted. In this regard, the researcher observed in the delivery room that one of the midwives said to a parturient woman: “Please pray for whatever you want, now God forgives you and accepts your prayers, please try to pray for those who want a baby, pray for us, too.” (A midwife, 35 years old, bachelor’s degree, work experience: 10 years)  

## Discussion

Some of the participants, based on information received from acquaintances, had cultural beliefs about normal childbirth. Those who favored cesarean section cited dysfunction in the performance of pelvic floor muscle, urinary incontinence, perianal relaxation and impairment in sexual pleasure as the side effects of vaginal delivery, which was an important reason to select C- section. In this regard, Klein’s study  (2012) about maternal request for cesarean in Canada showed that women, due to concerns about pelvic floor dysfunction, select cesarean section.^27^

The study of  Gungor (2008) in Turkey about the impact of mode of delivery on one’s sexual function showed that sexual performance and satisfaction is a mental issue and is more influenced by sociocultural beliefs than physical issues.^28^

Hence, it should be emphasized that vaginal delivery has no negative impact on the sexual performance of women in the future. This could create a positive mentality and beliefs in relation to natural childbirth and encourage women towards this type of delivery. 

In the present study, most of the participants considered C-section as an upper class “trend” and exclusive to the rich or people with a more stable economic status; this is mainly due to its higher cost compared to vaginal delivery. Surprisingly, a study shows that in Sweden, older women from lower social classes voluntarily chose C-section.^29^

Moreover, despite having financial problems, some pregnant women select C-section due to blind imitation. Gamble et al., who investigated the reasons for C-section preference, indicated that in most cases, medical norms of healthcare services were intertwined with non-medical matters.^30^

Wiklund (2006), in a study of the relationship between personality and mode of delivery, reported that in most cases older and lower class women choose cesarean section.^29^ Few sociological studies show that social class has an important role in pregnant women’s knowledge about various modes of delivery and their choices.^31^ Martin (2001) showed that social class has influence on women’s perceptions and expectations of their own body and mode of delivery.^32^

Thus, social values and norms related to the mode of delivery, including the social class of women, could have an impact on women’s perception of risk, and as a consequence on their choice of mode of delivery. So health professionals should encourage them to adopt healthy life styles and help them to gain skills and to learn effective problem-solving strategies to make better decisions about their mode of delivery.

In the present study, the participants believed that natural birth represents a woman’s vigor and power for earning the maternal role. Similarly, in other studies, women considered vaginal delivery as a life-changing experience and a natural process. Some women even felt a sense of failure if they could not give birth through vaginal delivery;^33^^,^^34^ these results are consistent with the findings of the present study.

Guaranteed maternal health following natural birth is one of the issues that present natural delivery as a safe delivery mode and a helpful way to improve family and public health. The main reason to choose natural delivery originates in the social beliefs related to the preference of natural birth due to its positive aspects for mother as well as lower mortality.

The results of a study in the United States showed that the participants believed in the safety of natural delivery compared to cesarean delivery due to reduced rate of delivery complications, such as postpartum bleeding and infection, and maternal death in cases of natural birth.^35^

Kasai (2010) studied women’s beliefs about delivery in Brazil and reported that belief in early recovery following normal birth is the main reason for giving priority to NVD.^36^ Aram et al. (2002), investigating the frequency of selected mode of delivery among pregnant women referred to health centers in Isfahan, Iran, found that the main reason for selecting NVD was lack of anesthesia side effects.^37^

It seems that in order to change social beliefs and traditions surrounding NVD, it is necessary to reinforce the positive cultural and social values towards this type of delivery and decrease the negative beliefs attached to it. The promotion of NVD as a safe childbirth method that improves maternal physical health could persuade the community to select natural birth more often. It could also create a positive atmosphere among pregnant women and enable them to make right decisions about their mode of delivery.

Local midwives presumed that cyclamen could relieve labor pain and facilitate the whole procedure. Detoxification during vaginal delivery is another benefit leading to the mother’s overall health. In a study by Sychareun et al. in Malaysia, the husband or the mother of the expectant woman was reported to provide her with money, napkins, baby’s clothes, ginger, rope, herbal medicine, boiled water, glove, soap, wood, and bamboo. A trained individual was responsible for providing the herbal medicines and ‘healing water’. They believed that these materials reduce labor pain, treat abnormal secretions, and strengthen the abdominal and uterine muscles for delivering the baby.^38^

Another cultural belief related to natural birth was closeness to God through giving birth naturally. Ajorpaz et al. (2012), in a clinical trial related to the impact of reading Holy Koran before C-section, found that Koran reading reduces stress in pregnant women.^39^

Bayrami et al. in their study (2011) of women’s experience of their first childbirth in Khoy and Marand, Iran, showed that reliance on God and Imams like *Imam Zaman* puts women in peace and serenity and increases their energy and hope, which in turn decreases their fear of NVD and enhances tolerance in women.^40^ In relation to fear, Abbaspour et al. (2014), studying women’s perception of factors influencing their fear of chilbirth, reported that  Iranian women’s fear of NVD plays an important role in their selection of a given birth method, which is cesarean section.^41^ Also, in the current study, each interviewee proposed her own suggestions to soothe or cope with delivery pain. Some of these suggestions are praying, promising to make offers to God, glorifying childbirth (considering a woman who dies during delivery a martyr), tying written prayers and Quran verses like ‘*Ayatolkorsi*’ around the mother’s foot during delivery, and paying more attention to spiritualities and less to metaphysical issues. Similarly, the results of the study by Ajorpaz et al. showed that Quran recitation could be effective for reducing stress in pregnant women.^39^

Considering the impact of cultural values on public perception of pregnancy, it is recommended these values be used for social recognition of NVD. Since behavioral changes based on religious beliefs are essential for providing a comfortable delivery for the believers, it is important to make these pregnant mothers familiar with rituals and spiritual issues before and during pregnancy. With respect to the influence of the Iranian culture on the views and perception of people regarding delivery, it is suggested efficient cultural processes based on ethical beliefs be used to promote NVD. 

This study used a qualitative approach to obtain first-hand data regarding the social attitudes towards delivery modes in Tonekabon, Iran. Maximum variation in sampling and the selection of different research environments for data collection were the advantages of the current study. Moreover, to our knowledge, no similar studies have been conducted on this subject in an Iranian cultural context. 

This study aimed to take a step towards changing socio-cultural beliefs and traditions about vaginal delivery by recognizing the common misconceptions about the definition and selection of this mode of delivery. Nevertheless, a number of limitations were noted in the current study: lack of enough motivation in some of the subjects for participating in the interviews and observations, and restricted access to local midwives due to their deaths. 

## Conclusion

The results of this study indicated that cultural beliefs significantly affect individuals’ attitudes towards modes of delivery, their definitions of these modes, and the choices they make. Social, cultural, and religious beliefs can determine how a woman perceives, interprets, and deals with pain during labor and how she selects pain management methods.

Positive attitudes towards this mode of delivery should be enhanced through various methods, and misconceptions should be corrected. 

The results of the present study showed that pregnant women’s decisions about mode of delivery have cultural, social and psychological dimensions. Understanding women’s views, experiences, preferences and social values related to mode of delivery help explain the decision making processes about mode of childbirth, decrease C-section rate and improve women’s health. To achieve this goal, it is necessary that policy makers, health planners, and managers of health systems and other social communities propose suitable strategies to change and encourage women towards NVD. The present results can help authorities to design culturally sensitive educational programs for mothers, husbands, local communities and gynecologists. Furthermore, the presentation of culture-based educational programs for women, as well as encouraging obstetricians and gynecologists to execute natural birth at lower costs, will contribute to the promotion of NVD. 
